# The Ecology of Stress: linking life-history traits with physiological control mechanisms in free-living guanacos

**DOI:** 10.7717/peerj.2640

**Published:** 2016-11-02

**Authors:** Ramiro J.A. Ovejero Aguilar, Graciela A. Jahn, Mauricio Soto-Gamboa, Andrés J. Novaro, Pablo Carmanchahi

**Affiliations:** 1 Laboratorio de Ecología Conductual, Instituto de Ciencias Ambientales y Evolutivas, Facultad de Ciencias, Universidad Austral de Chile, Valdivia, Chile; 2 Laboratorio de Interacciones Ecológicas, Instituto de investigaciones den zonas áridas (CONICET-MENDOZA-LIE-IADIZA), Mendoza, Argentina; 3 Grupo de Investigación de Eco-fisiología de Fauna Silvestre (GIEFAS), Asentamiento Universitario de San Martín de los Andes (AUSMA), Universidad Nacional del Comahue (INIBIOMA-CONICET-AUSMA-UNCo), Neuquén, Argentina; 4 Laboratorio de Reproducción y Lactancia, Universidad de Mendoza (IMBECU-CCT-MENDOZA), Mendoza, Argentina; 5 Programa Estepa-Andino Patagonica-CONICET-PATAGONIA NORTE-INIBIOMA, Neuquén, Argentina

**Keywords:** Stress ecology, Reproduction, Lama guanicoe, Sociality, Non-invasive methods, Hormonal profiles in wildlife

## Abstract

**Background:**

Providing the context for the evolution of life-history traits, habitat features constrain successful ecological and physiological strategies. In vertebrates, a key response to life’s challenges is the activation of the Stress (HPA) and Gonadal (HPG) axes. Much of the interest in stress ecology is motivated by the desire to understand the physiological mechanisms in which the environment affects fitness. As reported in the literature, several intrinsic and extrinsic factors affect variability in hormone levels. In both social and non-social animals, the frequency and type of interaction with conspecifics, as well as the status in social species, can affect HPA axis activity, resulting in changes in the reproductive success of animals. We predicted that a social environment can affect both guanaco axes by increasing the secretion of testosterone (T) and Glucocorticoid (GCs) in response to individual social interactions and the energetic demands of breeding. Assuming that prolonged elevated levels of GCs over time can be harmful to individuals, it is predicted that the HPA axis suppresses the HPG axis and causes T levels to decrease, as GCs increase.

**Methods:**

All of the data for individuals were collected by non-invasive methods (fecal samples) to address hormonal activities. This is a novel approach in physiological ecology because feces are easily obtained through non-invasive sampling in animal populations.

**Results:**

As expected, there was a marked adrenal (*p*-value = .3.4e−12) and gonadal (*p*-value = 0.002656) response due to seasonal variation in *Lama guanicoe*. No significant differences were found in fecal GCs metabolites between males/females*season for the entire study period (*p*-value = 0.2839). Despite the seasonal activity variation in the hormonal profiles, our results show a positive correlation (*p*-value = 1.952e−11, COR = 0.50) between the adrenal and gonadal system. The marked endocrine (*r*^2^ = 0.806) and gonad (*r*^2^ = 0.7231) response due to seasonal variation in male guanaco individuals highlights the individual’s energetic demands according to life-history strategies. This is a remarkable result because no inhibition was found between the axes as theory suggests. Finally, the dataset was used to build a reactive scope model for guanacos.

**Discussion:**

Guanacos cope with the trade-off between sociability and reproductive benefits and costs, by regulating their GCs and T levels on a seasonal basis, suggesting an adaptive role of both axes to different habitat pressures. The results presented here highlight the functional role of stress and gonad axes on a critical phase of a male mammal’s life—the mating period—when all of the resources are at the disposal of the male and must be used to maximize the chances for reproductive success.

## Introduction

Natural selection shapes phenotypic traits that permit organisms to respond appropriately to intrinsic and extrinsic factors (Darwin, 1959; Roff, 1992; Stearns, 1992; Bozinovic, 2002; Nespolo, Bacigalupe & Bozinovic, 2003; Clutton-Brock et al., 2001). Phylogeny and genetic variation among organisms determine the resource allocation trade-offs among life-history traits (e.g. age-specific growth, reproduction and survival schedules, and sociability). Environmental, ecological, and demographic processes (extrinsic factors) create selective pressures that impinge on an organism’s lifetime fitness (Stearns, 1992; Zera & Harshman, 2001). Therefore, understanding life-history variation among taxa from an evolutionary perspective requires an understanding of the physiological mechanisms that link genes with phenotype and how extrinsic factors regulate trade-offs between survival and reproduction. Furthering our knowledge of ecophysiological mechanisms, and particularly endocrine mechanisms, is key to understanding how life-history variation and trade-offs arise and are retained (Ketterson & Nolan Jr, 1999; Zera & Harshman, 2001; Crespi et al., 2013; Becker, 2002; Von Holst, 1998; Jacobs & Wingfield, 2000).

The stress response is a mechanism by which an individual of a given species copes with environmental changes (stressors) (Boonstra, McColl & Karels, 2001; Wingfield, 2005; Romero, Dickens & Cyr, 2009; Ovejero & Carmanchahi, 2012; Ovejero, 2013; Boonstra et al., 2007). Perception of a stressful situation activates the sympathetic adrenomedullary system and the hypothalamic pituitary adrenocortical (HPA) axis (Mostl & Palme, 2002; Busch & Hayward, 2009; Sapolsky, Romero & Munck, 2000). Activation of the HPA axis stimulates the release of steroid hormones (glucocorticoids GCs, e.g., corticosterone, cortisol). GCs in turn activate the mobilization of energy necessary to cope with adverse environmental conditions (Dallman et al., 2007; Sapolsky, Romero & Munck, 2000).

In the last 30 years, the term stress has been strongly criticized due to its ambiguity and several authors have researched the allostasis theory and attempted to connect biomedical and ecological data (McEwen & Wingfield, 2003; Romero, Dickens & Cyr, 2009; Romero, Dickens & Cyr, 2009. Allostasis is the daily and seasonal process that allows organisms to maintain physiological parameters (homeostasis) within life-sustaining ranges, which occur via allostatic mediators (McEwen & Wingfield, 2003). The allostatic model generated criticism and Romero, Dickens & Cyr (2009) proposed the reactive scope model, attempting to retain the strengths but avoid the weaknesses of the allostatic model. The present work contributes to the debate about the applicability of the allostatic model to nature.

Extrinsic factors, like anthropogenic disturbance, biological invasions, or climate change (in terms of allostasis) can negatively affect behavior, reproduction, immune function and growth through prolonged activation of the HPA axis (Moberg, 1991; Boonstra & Singleton, 1993; Goymann & Wingfield, 2004; Pride, 2005; Pereira, Duarte & Negrão, 2006). Glucocorticoids (GCs) are often considered the allostatic mediators of the stress response, and they have been the focus of research for decades (Sapolsky, Romero & Munck, 2000; Ovejero, 2013; Cavigelli, 1999; Levine, 2005; Möstl, Rettenbacher & Palme, 2005; Raouf et al., 2006; Romero, 2004; Romero & Remage-Healey, 2000; Sheriff et al., 2009; Sheriff et al., 2011; Smith et al., 2012; Soto-Gamboa et al., 2009; Tarlow & Blumstein, 2007; Touma & Palme, 2005; Vera, Zenuto & Antenucci, 2012; Wasser et al., 2000; Wingfield & Sapolsky, 2003). Much less is known, however, about the role of GCs’ in free-living animals (Bonier et al., 2009). The role of baseline or stress-induced GC in the modulation of an individual or populations performance, is still essentially unknown. Understanding, social factors and their endocrine correlates (baseline measures of GCs and T) in wild animals has potential implications for wildlife management, population ecology, reproductive biology and evolution. The social environment is therefore one of the primary sources of information that can induce allostatic responses (Boonstra, McColl & Karels, 2001; Rubenstein & Shen, 2009; Creel et al., 2013; Blanchard, McKittrick & Blanchard, 2001). It’s expected that in both social and non-social animals, the frequency and type of interaction with conspecifics, as well as status in social species,can affect HPA and HPG axis activity and ultimately the reproductive success of animals.

During the breeding season of many mammals, males commonly increase testosterone levels in response to social stimuli such as presence of male competitors or potential mates (Enstrom, Ketterson & Nolan Jr, 1997; Cavigelli & Pareira, 2000; Sinervo et al., 2000; Ostner & Kappeler, 2002; Soto-Gamboa, Villalon & Bozinovic, 2005; Schradin, 2008; Creel et al., 2013). This physiological mechanism (androgen response) modulated by social environment, is likely to be mainly important when considering their roles (testosterone-mediated traits) in the evolution of mating systems and life histories (Wingfield et al., 1997; Knapp & Moore, 1997; Faulkes & Abbott, 1997; Hirschenhauser & Oliveira, 2006; Young et al., 2006; Rubenstein & Shen, 2009; McGlothlin et al., 2010; Creel, 2001; Lofts & Marshall, 1960). In addition, testosterone levels can also affect female preference (Enstrom, Ketterson & Nolan Jr, 1997), thus influencing fitness through both intra and intersexual selection; (Sinervo et al., 2000). Although the role of short-term androgen changes has not been completely described, these have long been associated with territoriality (McGlothilin et al., 2010). The “Challenge Hypothesis” (first described in bird studies) predicts that androgen levels and agonistic interactions are positively related and are socially-context dependent (Wingfield et al., 1997). Among mammals, these predictions have recently been tested, and a positive correlation was found between social cues and testosterone levels associated with territorial defense, group formation, social dominance and hierarchy formation during the breeding season (Muller & Wrangham, 2004; Creel et al., 2013). Consequently, social stressors provide a context where the individual must respond to a series of repeated stressors over time, depending on the frequency and intensity of the stimulus, and this can lead to various physiological problems, (see Sapolsky, 2002; Côte, 2000).

Much of the variation in life histories strategies reflects individuals’ phenotypic responses to environmental challenges and perceived risks. The adrenal and gonadal system strongly influence behavior, control the annual cycle of development, modulate behavioral and physiological responses to the environment, and establishes important incompatibilities in life stages. It provides a model for studying the connections between mammal physiology and life history. Guanacos (*Lama guanicoe*) are the largest social artiodactyls of South America, and these organisms have a polygynous resource-defense mating system. This wild and endemic camelid has been successfully used as a study model of behavioral interactions De Lamo et al., 1998; Franklin, 1983; Marino & Baldi, 2008; Taraborelli et al., in press ecological studies (Puig & Videla, 1995; Bank, Sarno & Franklin, 2003; Puig et al., 2008; Acebes et al., 2009; Ovejero et al., 2011; Ovejero, 2013; Marino & Baldi, 2014; Radovani et al., 2004; Puig & Rabinovich, 1995; Raedeke, 1979; Young & Franklin, 2004) and management (Montes et al., 2005; Bonacic & Macdonald, 2003; Zapata et al., 2004), but information about the guanaco’s physiological ecology remains scarce. More recently, guanacos have also been used as a model of stress response; a study was conducted to determine how management including handling, shearing, and release induce changes in circulating steroid hormones (Carmanchahi et al., 2011); however, this work was based on an invasive sampling method.

To understand how physiology mediates the relationship between life history and the environment, we predict that: (A) Social environment positively will affect the HPG and HPA axes by raising the secretion of T and GCs due to individual social interactions and the energetic demands of the breeding season; (B) Male guanacos will show seasonal variation in the activity of their adrenal and gonadal systems, which perform incompatible functions in different life stages (survival vs reproduction); (C) Prolonged elevated baseline levels of GCs in male guanacos due to long periods of intense social interaction will be detrimental, costly, and decrease fitness, and we would expect that in this case HPA inhibits the activity of the HPG. Together, these predictions provide a description of the reactive scope model and an understanding of how physiological mechanisms might constrain patterns of variation in wild South-American camelid life histories. These predictions might also provide the basis for future mammalian studies targeted at understanding phenotype-environment interactions.

## Methods

### Ethics statement

The present study did not warrant capturing or handling protected or endangered animals. All of the data for individuals were collected by non-invasive methods (fecal samples) to address hormonal activities. This is a novel approach in physiological ecology because feces are easily obtained through non-invasive sampling in animal populations. This is important because the handling necessary for collection of plasma can confound estimates of baseline GC measurements. The described field studies were carried out in a protected area with a permit (files:NO 4350-000019112/Res 238) from the DRNR (Dirección de Recursos Naturales Renovables, Mendoza).

### Non-invasive methods to address hormonal activities

Fresh fecal samples were collected from 334 wild adult male guanacos from La Payunia reserve (36.000S and 36.360S; 68.340W and 69.230W, South of Mendoza Province, Argentina). The samples were collected during 7 field surveys (15 days each month) over the course of a year. Fresh feces samples were collected from focal individuals (one sample per each individual) and were labeled according to the sample location, time, sex, age and social structure (family group, bachelors group and solitary male). To slow microbial activity and to reduce immunoreactivity problems, the samples were frozen immediately in liquid nitrogen and stored at −20°C until the time of analysis (Laboratorio de reproducción y lactancia-LARLAC-IMBECU-CONICET-MENDOZA). Fieldwork was conducted during the breeding and non-breeding months to assess seasonal variation in hormone levels. We choose this period (from September to March) because during the beginning of the breeding season, guanaco males are exposed to intense social instability caused by agonistic interactions with other males due to territory establishment and hierarchies (Ovejero, 2013) and at the end males are exposed to female defense interactions. This suggests that for guanaco males, female or territory defense strategies demand higher energetic costs. This is expected if one assumes that defending a female or territory are the principal objective (and target) of a male that wants to maximize the probabilities of leaving an offspring. We expected that this behavioural expression have physiological mechanism that could explain this difference. During the non-breeding season (from April to August), a period with less intense social interactions, we expected lower activity on hormonal mechanism.

Furthermore, 100% of the sampling surveys were done in the same sites. Steroids were extracted from lyophylized fecal samples according to a protocol developed for fecal steroid metabolite extraction for free-range ungulates (for more detail, see Ovejero, 2013; Buchanan & Goldsmith, 2004; Mateo & Cavigelli, 2005). The commercial^125^I-cortisol and ^125^I-testosterone RIA-KITS (BECKMAN, Coulter Company IM-1841/IM-1119-Immunotech-, Prague, Czech Republic) were used to quantify the levels of cortisol and testosterone in the samples (for more detail, see Ovejero, 2013). The analytical sensitivity of the cortisol assay is 3.075 ng/mL. The antibody used in the immunoassay is highly specific for cortisol (100% cross-reaction). The cross-reactivity to other naturally occurring steroids is very low, for example: aldosterone <0.1%; 8.4% corticosterone; cortisone 1.5%; 11-desoxy-cortisol 18%, etc. The analytical sensitivity of the testosterone assay is 0.025 ng/mL. The antibody used in the immunoassay is highly specific for testosterone (100% cross-reaction). The cross-reactivity to other naturally occurring steroids is very low, for example: 0.00014% aldosterone; estrone <0.3%; 0.08 androsterone; 5-Dihydrostestosterone 10% *α*; 11*β*-Hydroxytestosterone 2%; corticosterone 8.4%; 1.5% cortisone; cortisol 11-deoxy-18%, etc. In addition, all samples were assayed in triplicate (extraction efficiency test) and duplicate (binding and parallelism test) and reanalyzed whenever the resulting coefficient of variation exceeded 20%. Intra- and inter-assay coefficients of variation of GCs-immunoassay were 6.5% and 11.2% and for testosterone immunoassays were 9.5 and 13.4%, respectively.

### Statistical analyses

Welch two sample T-tests were used to evaluate the seasonal variation response of steroid hormones. Furthermore, two-way ANOVAs were used to evaluate the interaction between sex and season. Generalized linear models were developed (*n* polynomials function =*f*(*x*) = *a*_*n*_*X*^*n*^ + *a*_*n*1−1_*X*^*n*^ − 1 + *a*_*n*−2_*X*^*n*^ − 2 + …) to describe the hormonal profiles and their seasonal variation in response to the environment. Moreover, a Pearson’s correlation was used to test the activity association between the adrenal and gonadal system throughout the year. All statistical analyses were conducted using R statistical software version 3.1.0 (R Development Core Team, 2012).

## Results

### Ecological process and fecal-hormone profiles in wild guanacos

**Figure 1:**
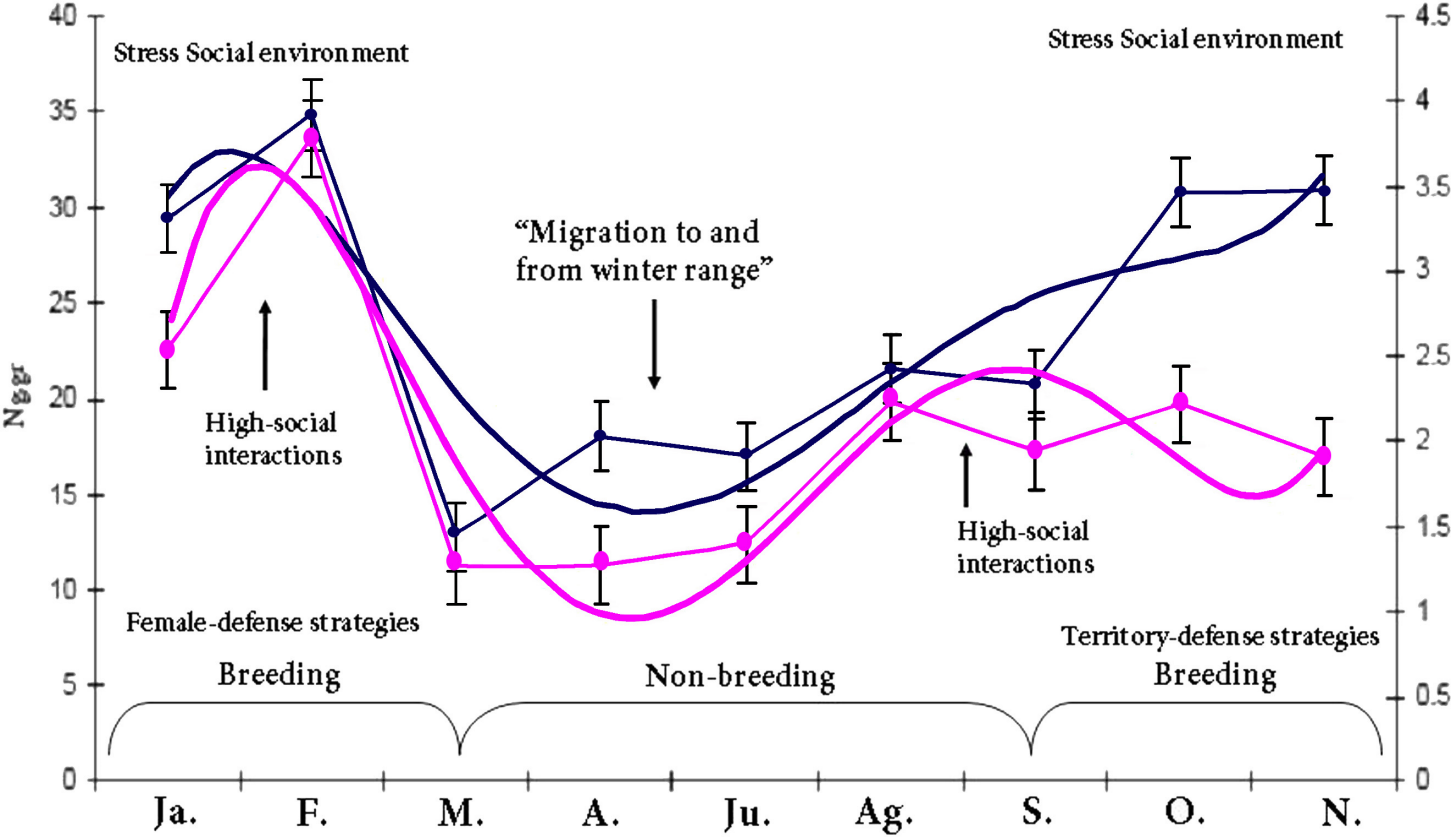
HPA & HPG axis activity patterns throughout the study period. Blue line, the seasonal baseline fecal testosterone profile (defined by *y* = 0.0041*x*5 − 0.1102*x*4 + 1.0889*x*3 − 4.6819*x*2 + 7.914*x* − 0.7892; *r*^2^ = 0.7231), Pink line, the seasonal baseline fecal cortisol profile (defined by *y* = 0.0743*x*5 − 1.9677*x*4 + 19.136*x*3 − 82.442*x*2 + 147.4*x* − 58.902; *r*^2^ = 0.806). There was marked adrenal and gonadal activity during the breeding and non-breeding (CI = 7.661734–12.057391, mean = 25.75864/15.89907 respectively) season respectively for the male groups, the GCs and T response was due to seasonal variation in the guanacos highlighting the individual’s energetic demands according to life-history strategies.

**Figure 2:**
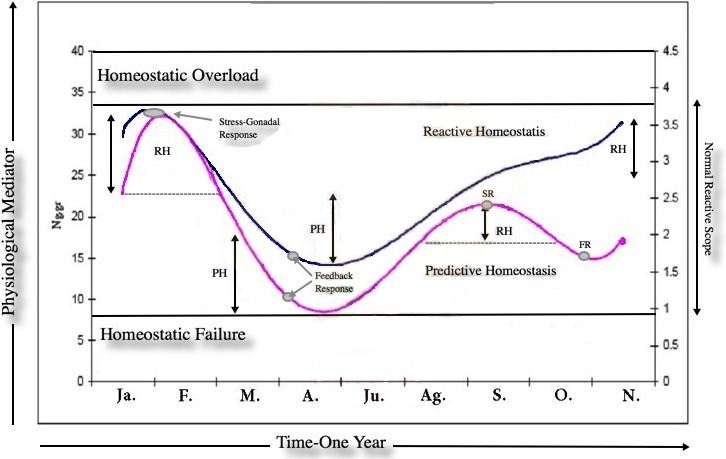
The reactive scope model for guanacos. Most studies measure only one physiological mediator at a time, here the concentration of two mediators (cortisol-C /testosterone.T) is placed on the *y*-axis at a given time point. The mediators are correlated in terms of the functional role that each one plays in the normal reactive scope range. In other words, both mediators encompass responses for coping with predictable and unpredictable changes in the environment. The values of each mediator are presumed to exist in four general ranges. The minimun (5–10 Ng.gr for C; 1–2 Ng.gr for T) and maximun (20–35 Ng.gr for C; 3–4 Ng.gr for T) concentration of the mediator was presumed to constitute a threshold. The results show the ranges (double arrows) of predictive/reactive homeostasis (PH/RH) and homeostatic overload/failure, the stress-gonadal and feedback response (gray circles) indicates the max-min activity patterns for HPA & HPG axis during one year. The C activity patterns in guanacos shows two peak of RH (dotted lines) in the reproductive season, between Ag./O. males adopts a territory-defense strategies at the beginning of the reprod. season; during Ja./F. males changes to a female-defense strategies at the ends of the reprod. season. These results consist of the seasonal set of point ranges for the physiological mediators.

As expected, there was a marked adrenal (*t* = 7.4016, *df* = 302.174, *p*-value = 1.344e−12, 1-*α* (0.90) = 7.661734–12.057391, mean = 25.75864/15.89907 breeding and non-breeding season respectively) and gonadal (*t* = 3.2873, *df* = 28.948, *p*-value = 0.002656, 1-*α*(0.90) = 0.6213–1.9508, mean = 3.209810/1.923700 breeding and non-breeding season respectively for male groups) GCs and T response due to seasonal variation in *Lama guanicoe* individuals. This highlights the individual’s energetic demands according to life-history strategies. No significant differences were found in the amounts of fecal GC metabolites between males/females*season for the entire study period (*F* = 1.0757, *df* = 143.529, *p*-value = 0.2839; male: ng/gr = 22,42,1-*α* (0.90) = 1.6887, std-e = 0.86, *n* = 251; female:ng/gr = 20,71,1-*α* (0.90) = 2.6079, *n* = 76, std-e = 1.33). This result was not expected as it was thought that there would be a difference in the allocation of resources to cope with the different roles that that males and females have in a population. Despite the seasonal activity variation in the hormonal profiles, our results show a positive correlation (*t* = 7.1524, *df* = 184, *p*-value = 1.952e−11, CI = 0.3457400 0.5719168, COR = 0.50) between the adrenal and gonadal system; both peaks of activity concur with the reproductive season. Our last prediction states that prolonged elevated baseline levels of GCs due to long periods of intense social interaction are detrimental, costly and decrease fitness; it is expected that in this context, HPA inhibits the activity of the HPG. The marked endocrine (*y* = 0.0743*x*5 − 1.9677*x*4 + 19.136*x*3 − 82.442*x*2 + 147.4*x* − 58.902; *r*^2^ = 0.806) and gonad (*y* = 0.0041*x*5 − 0.1102*x*4 + 1.0889*x*3 − 4.6819*x*2 + 7.914*x* − 0.7892; *r*^2^ = 0.7231) response due to seasonal variation in male guanaco individuals (Fig. 1) highlights the individual’s energetic demands according to life-history strategies. This is a remarkable result because no inhibition was found between the axes as theory suggests. Finally, the dataset was used to build a reactive scope model for guanacos, see Fig. 2. Most studies measure only one physiological mediator at a time; here the concentration of two mediators is placed on the *y*-axis for a given time point. This was done because these mediators are correlated in the functional role that each one plays in the normal reactive scope range. In other words, both mediators encompass responses to cope with predictable and unpredictable changes in the environment. Our result shows the ranges of predictive/reactive homeostasis and homeostatic overload/failure. These ranges consist of the seasonal set of point ranges for the physiological mediators. The mediators are correlated in terms of the functional role that each one plays in the normal reactive scope range. In other words, both mediators encompass responses for coping with predictable and unpredictable changes in the environment. The values of each mediator are presumed to exist in four general ranges. The minimum (5–10 Ng.gr for C; 1–2 Ng.gr for T) and maximum (20–35 Ng.gr for C; 3–4 Ng.gr for T) concentration of the mediator was presumed to constitute a threshold. The results show the ranges of predictive/reactive homeostasis (PH/RH) and homeostatic overload/failure, the stress-gonadal and feedback response indicates the max–min activity patterns for HPA & HPG axis during one year. These results consist of the seasonal set of point ranges for the physiological mediators.

## Discussion

Our results confirm the prediction that “Social environment positively affects the HPG and HPA axes by raising the secretion of T and GCs due to individual social interactions and the energetic demands of the breeding season.” Further to this, our results suggest that social environment and breeding season are stressful scenarios for male guanacos. These scenarios are seen to produce associated costs such as increased metabolic rate and energy expenditure due to: territorial/female defense (male-reproduction strategies), male social dominance hierarchies, reproduction, and increased exposure to predators during the breeding season. If the data value observed in March is taken as a baseline cortisol level, cortisol is seen to increase by one and a half times during the non-breeding season and three times during the breeding season. If the March testosterone measurement is taken as the basal testosterone level, the same pattern is seen; the hormonal profile increases during the non-breeding and breeding seasons. This highlights the importance of the functional roles of the HPA and HPG axes during the reproductive period, as well as the adaptive roles they play in the seasonal pattern observed, allowing the species to meet environmental challenges as well as challenges throughout its life cycle.

From the results gathered in this study, different inmunoreactive GCs and T secretion patterns were observed according to the mating system and reproduction strategies (territorial defense and female defense) that guanaco males (of a migratory population) adopt during the breeding season (Ovejero, 2013). Our results highlight the functional role of the stress and gonad axes during the critical mating period when all of the resources at a male’s disposal must be used to maximize the chances of reproductive success. Generally, it appears that T is immediately involved in aggression associated with reproduction, rather than with other forms of aggression (e.g., anti-predator aggression, Moyer, 1968; Marler et al., 1988). The data here presented support the correlation between T patterns and reproductive strategies (establishment and maintenance of a breeding territory and mate-guarding) proposed by Wingfield et al. (1990) for male birds. However, this study extends the findings of Wingfield et al. (1990) to mammals. In 1984, the same authors proposed that males of polygynous species may have higher levels of T for longer periods during the breeding season than do males of monogamous species. Our results highlight the functional role that T levels have in determining and maintaining the polygynous mating system in guanacos, but we predict that this pattern could be altered in guanaco populations that coexist with human activities that fragment the guanaco’s normal distributional range. For example, the use of fences by rural settlers has a direct impact on the space used by guanacos, and it is possible that this could promote some groups (within a population) to adopt monogamous behavior (Marino & Baldi, 2008).

On the other hand, our results highlight the notorious decrease in the activity pattern of the stress and gonadal axes during the non-breeding season. This result was not expected because individuals must cope with another critical phase of autumnal migration at this time, therefore we expected that all of the resources (in terms of energy) would be available to maximize the chances for survival success during the migration trip. These results confirm our prediction that “guanacos show a seasonal variation in the activity of their endocrine and gonadal systems and incompatible functions (reproduction an self-maintenance) are established during different life stages.” In winter, food intake by birds and mammals needs to be geared towards preventing starvation while at the same time maintaining an optimum body mass to permit rapid escape from predators. GC concentrations may be the critical factor determining the size of the fat reserves and either environmental severity or predation risk (Wingfield et al., 1990). The GC hormone, corticosterone, is thought to be important in the physiological orchestration of avian migration because of the identified elevated level of activity of the avian adrenal gland either prior to or during migration (Naik & George, 1963; John, 1965; Peczely, 1976). Preparation for migration and for winter requires a period of hyperphagia and a laying down of fat reserves (for a review, see Holberton, 1999). In birds, Wingfield & Silverin (1986); Astheimer, Buttemer & Wingfield (1992) and mammals, Dallman et al. (1993), GCs play a vital role in foraging behavior and hyperphagia, with low to moderate concentrations stimulating feeding behavior. As is noted by O’Reilly & Wingfield (1995) migrating birds show increased baseline concentrations of GCs but a reduced stress response. An adaptive explanation for this pattern is the migration modulation hypothesis, Holberton, Parrish & Wingfield (1996) and Holberton (1999). It proposes that higher than average concentrations of baseline GCs are expressed prior to and during migration to facilitate migratory fattening. However, the stress response is lowered when challenged so that the negative, catabolic effects of high GCs on skeletal muscle do not occur; thus, critical skeletal muscles are preserved for flight during migration. As such, this hypothesis may explain the non-breeding season hormonal profiles generated in this study, but more data is needed to test these assumptions. Another explanation may suggest that migration (in terrestrial system) could be regulated by other endocrine mechanisms (and other hormones) involved in generating fat reserves and foraging behavior such as those that involve the thyroid gland Schwabl, Wingfield & Farner (1984). Mattocks (1976) shows that gonad hormones (at least in some species) have a functional role (fat deposition) before spring migration. This may suggest that the increased activities of the gonad axis compared with the stress axis in guanacos could have an important role in the “migration to and from the winter range”, but to confirm this prediction more experimental and field studies would be needed.

The last prediction of this study, that “prolonged elevated baseline levels of GCs due to long periods of intense social interaction will be detrimental, costly and decrease fitness” was not validated by the results gathered. Our interest in the relationships between social or environmental conditions and stress-gonadal hormone concentrations (for both social and non-social species) is motivated by a desire to understand the physiological mechanisms by which the environment affects fitness. However, understanding the relationship between hormonal profiles and fitness is not straightforward, therefore this study attempts to obtain proxies that would help us begin to resolve this relationship. We consider that taken together, the interplay between stress/gonad hormones across seasonal variation could be an interesting proxy to “establish” causal relationships between hormonal levels and ecological correlates. For example, some authors have stated that prolonged elevated baseline levels of GCs are commonly viewed to be detrimental, costly and decrease fitness (Bonier et al., 2009). However, there is scarce evidence for this negative association. Part of the problem with this approach is the variation among species in terms of their hormones/fitness relationships, and this variation can be attributed to a variety of demographic, social (e.g., social rank, age, sex, group size) or environmental factors (e.g., resource availability). Additionally, for a variety of vertebrate taxa, seasonal variation in GC levels has been reported to be due to varying food availability, temperature, rainfall and tourist activity (Romero, 2002; Tempel & Gutierrez, 2004; Kenagy & Place, 2000); however, it still remains unclear if this holds true for mammals. The other issue is that many of the studies of HPA-fitness relationships reviewed by Bonier et al. (2009) were conducted during the breeding season alone. The study herein presented provides a full set of data (hormone profiles) across seasonal variation throughout the course of an entire year. As expected, guanacos cope with this trade-off between sociability and reproductive benefits and costs by regulating their GCs and testosterone levels on a seasonal basis like other mammals (Boonstra, 2005), and they may also experience fine adjustments on a daily basis. We suggest that elevated levels of GCs in guanacos due to highly social interactions during the breeding season may play a fundamental role in the regulation of enhanced metabolic needs during reproduction. Guanaco males, like other iteroparous males, exhibit high concentrations of cortisol, a gonadal axis that is not inhibited by high GC concentrations, so the last prediction presented in this work must be rejected. Furthermore, during the breeding season, our results show two GC peaks of activity, one at the beginning and another at the end of the breeding season. In the former, guanaco males are exposed to intense social instability caused by agonistic interactions with other males due to territory establishment and hierarchies. In the latter, guanaco males are exposed to female defense interactions. This suggests that for guanaco males, female defense strategies demand higher energetic costs when compared to territory defense strategies. This is expected if one assumes that defending a female (rather than a territory) is the principal objective (and target) of a male that wants to maximize the probabilities of leaving an offspring. Moreover, birth rate data for the population was collected from 2007–2008 (hormonal measures)-2009, so if one expects a negative relationship between the hormonal profiles, one would also expect to see a negative value for the proportion of offspring per adult. The birth rate values for those years were 0.14, 0.28 and 0.29 respectively; thus with these results one can expect that there is no detrimental effect on individual-population fitness.

The lesser HPA axis activity measured during the non-breeding season confirms the prediction “that reproduction and sociability may be long-term and predictable.” This is likely the case because this period is associated with fewer social interactions. The individual hierarchies are more relaxed because the animals are preparing to migrate (A Novaro, pers. comm., 2015) and the species strategy at this time is to survive and to not reproduce.

Finally from the data collected here, a reactive scope model for guanacos has been generated. The results show the ranges of predictive/reactive homeostasis and homeostatic overload/failure ranges; these consist of the seasonal set of point ranges for the physiological mediators. For guanacos, the normal reactive scope is described with two GCs and T set point ranges. The model was made to reflect the natural progression of life-history stages such as breeding. Furthermore, the minimum concentration of the mediator was presumed to constitute a threshold. Subsequently, a GC and T threshold was proposed, and below this limit it is seen that the individual/population enters into homeostatic failure. This threshold is predicted to indicate the minimum concentration of GCs and T mediator necessary to sustain guanacos seasonal life changes (in natural conditions). However, more studies are needed to test this prediction. On the other hand, above the upper end of the normal reactive scope, when a physiological mediator exceeds the reactive homeostatic range, the individual/population enters into homeostatic overload (pathological states). This threshold is predicted to indicate the maximum concentration of GCs and T mediator necessary to sustain seasonal life changes in guanacos (in natural conditions). However, more studies are needed to test this prediction especially considering that pharmacological stimulation may allow individuals to reach a higher threshold though this would not be considered within the scope of natural changes.

## Conclusions

Knowledge of physiological and behavioral mechanisms is key to understanding how life-history variation and trade-offs might arise and help organisms cope with environmental variation. Mechanistically, trade-offs result from the need to differentially allocate limited resources to traits like reproduction versus self-maintenance, with selection favoring the evolution of optimal allocation mechanisms. Guanacos cope with the trade-off between sociability and reproductive benefits and costs by regulating their GCs and T levels on a seasonal basis. This seasonal regulation suggests the adaptive and functional role of both axes in regulating energy allocation for predictable life-history events (like reproduction). During breeding, and event with highly social interactions, elevated baseline levels of GCs and T may actually be predictive of high reproductive success. From this, we propose that guanacos anticipate stressors with acute impacts and the duration is moderated by physiological consequences (i.e. reproduction). During the non-breeding season, a period with less intense social interactions, lower baseline levels of GCs and T may actually be predictive of a high probability of survival. Our study provides a model for further study of the links between physiology and life history.

Studying both the HPA and the HPG axes was an informative approach to understand the functional mechanisms that animals use to cope with stress. Studying these two axes simultaneously is important because the key circulating steroid hormones of the stress axis, GCs, influence the expression of approximately 10% of the genome. Some of the targets of GCs include genes controlling metabolism, growth, repair, reproduction, and management of resource allocation (Le et al., 2005). However, both axes play fundamental roles not just when animals are experiencing stress, but also during normal periods of survival and growth. Therefore, these axes mediate the adaptation of the organism to its environment. At the individual level, the stress axis plays a key role in allowing animals to respond to the changes and challenges of both environmental certainty and uncertainty. At the species level, the stress axis plays a central role in evolutionary adaptation to particular ecological pressures.

##  Supplemental Information

10.7717/peerj.2640/supp-1 Data S1 Raw data

## Additional Information and Declarations

### Field Study Permissions

The following information was supplied relating to field study approvals (i.e., approving body and any reference numbers): The described field studies were carried out in a protected area with a permit (files:NO 4350-000019112/Res 238) from the DRNR (Dirección de Recursos Naturales Renovables, Mendoza).

### Data Availability

The following information was supplied regarding data availability: The raw data has been supplied as a Data S1

## References

[ref-1] Acebes P, Traba J, Malo JE, Ovejero R, Borghi CE (2009). Density and habitat use of an isolated population of the guanaco (Lama guanicoe) in the Monte desert of Argentina. Mammalia.

[ref-2] Astheimer LB, Buttemer WA, Wingfield JC (1992). Interactions of corticosterone with feeding, activity and metabolism in passerine birds. Ornis Scandinavica.

[ref-3] Bank MS, Sarno RJ, Franklin WL (2003). Spatial distribution of guanaco mating sites in southern Chile: conservation implications. Biological Conservation.

[ref-4] Becker JB (2002). Behavioral endocrinology.

[ref-5] Blanchard RJ, McKittrick CR, Blanchard DC (2001). Animal models of social stress: effects on behavior and brain neurochemical systems. Physiology and Behavior.

[ref-6] Bonacic C, Macdonald DW (2003). The physiological impact of wool-harvesting procedures in vicunas (Vicugna vicugna). Animal Welfare-Potters Bar then Wheathampstead.

[ref-7] Bonier F, Moore IT, Martin PR, Robertson RJ (2009). The relationship between fitness and baseline glucocorticoids in a passerine bird. General and Comparative Endocrinology.

[ref-8] Boonstra R (2005). Equipped for life: the adaptive role of the stress axis in male mammals. Journal of Mammalogy.

[ref-9] Boonstra R, Barker JM, Castillo J, Fletcher QE, Wolff JO, Sherman PW (2007). The role of the stress axis in life-history adaptations of rodents. Rodent societies: an ecological and evolutionary perspective.

[ref-10] Boonstra R, McColl CJ, Karels TJ (2001). Reproduction at all costs: the adaptive stress response of male arctic ground squirrels. Ecology.

[ref-11] Boonstra R, Singleton GR (1993). Population declines in the snowshoe hare and the role of stress. General and Comparative Endocrinology.

[ref-12] Bozinovic F (2002). Physiological ecology and evolution. Theory and study cases in vertebrates.

[ref-13] Buchanan KL, Goldsmith AR (2004). Noninvasive endocrine data for behavioural studies: the importance of validation. Animal Behavior.

[ref-14] Busch DS, Hayward LS (2009). Stress in a conservation context: a discussion of glucocorticoid actions and how levels change with conservation-relevant variables. Biological Conservation.

[ref-15] Carmanchahi P, Ovejero RJA, Mar ull C, López C, Schroeder N, Jahn G, Novaro AY, Somoza G (2011). Physiological response of wild guanacos to capture for live shearing. Wildlife research.

[ref-16] Cavigelli SA (1999). Behavioural patterns associated with faecal cortisol levels in free-ranging female ringtailed lemurs, *Lemur catta*. Animal Behavior.

[ref-17] Cavigelli S, Pareira M (2000). Mating season aggression and fecal testosterone levels in male ring-tailed lemurs (Lemur catta). Hormones and Behavior.

[ref-18] Clutton-Brock TH, Brotherton PNM, Russell AF, O’Riain MJ, Gaynor D, Kansky R, Griffin A, Manser M, Sharpe L, McIlrath GM, Small T, Moss A, Monfort S (2001). Cooperation, control, and concession in meerkat groups. Science.

[ref-19] Côte SD (2000). Dominance hierarchies in female mountain goats: stability, aggressiveness and determinants of rank. Behaviour.

[ref-20] Creel S (2001). Social dominance and stress hormones. Trends in Ecology & Evolution.

[ref-21] Creel S, Dantzer B, Goymann W, Rubenstein DR (2013). The ecology of stress: effects of the social environment. Functional Ecology.

[ref-22] Crespi EJ, Williams TD, Jessop TS, Delehanty B (2013). Life history and the ecology of stress: how do glucocorticoid hormones influence life-history variation in animals?. Functional Ecology.

[ref-23] Dallman MF, Strack AM, Akana SF, Bradbury MJ, Hanson ES, Scribner KA, Smith M (1993). Feast and famine: critical role of glucocorticoids with insulin in daily energy flow. Frontiers in Neuroendocrinology.

[ref-24] Dallman MF, Warne JP, Foster MT, Pecoraro NC (2007). Glucocorticoids and insulin both modulate caloric intake through actions on the brain. Journal of Physiology.

[ref-25] Darwin C (1959). On the origin of species by means of natural selection, or the preservation of favoured races in the struggle for life.

[ref-26] De Lamo D, Sanborn A, Carrasco C, Scott D (1998). Daily activity and behavioral thermoregulation of the guanaco (Lama guanicoe) in winter. Canadian Journal of Zoology.

[ref-27] Enstrom DA, Ketterson ED, Nolan Jr V (1997). Testosterone and mate choice in the dark-eyed junco. Animal Behavior.

[ref-28] Faulkes CG, Abbott DH, Solomon NG, French JA (1997). The physiology of a reproductive dictatorship: regulation of male and female reproduction by a single breeding female in colonies of naked mole-rats. Cooperative Breeding in Mammals.

[ref-29] Franklin W, Eisenberg JF, Kleiman D (1983). Constranting socioecologies of South America’ s wild camelids: the vicuna and guanaco. Advances in study of mammalian behavior. Special Publications No. 7.

[ref-30] Goymann W, Wingfield JC (2004). Allostatic load, social status and stress hormones: the costs of social status matter. Animal Behavior.

[ref-31] Hirschenhauser K, Oliveira RF (2006). Social modulation of androgens in male vertebrates: meta-analyses of the challenge hypothesis. Animal Behaviour.

[ref-32] Holberton RL (1999). Changes in patterns of corticosterone secretion concurrent with migratory fattening in a Neotropical migratory bird. General and Comparative Endocrinology.

[ref-33] Holberton RL, Parrish JD, Wingfield JC (1996). Modulation of the adrenocortical stress response in Neotropical migrants during autumn migration. The Auk.

[ref-34] Jacobs JD, Wingfield JC (2000). Endocrine control of life- cycle stages: a constraint on response to the environment?. The Condor.

[ref-35] John TM (1965). A histochemical study of adrenal corticoids in pre and post migratory wagtails, *Motacilla alba* and *M. flava*. Pavo.

[ref-36] Kenagy GJ, Place NJ (2000). Seasonal changes in plasma glucocorticosteroids of free- living female yellow-pine chipmunks: effects of reproduction and capture andhandling. General and Comparative Endocrinology.

[ref-37] Ketterson ED, Nolan Jr V (1999). Adaptation, exaptation, and constraint: a hormonal perspective. American Naturalist.

[ref-38] Knapp R, Moore MC (1997). Male morphs in tree lizards have different testosterone responses to elevated levels of corticosterone. General and Comparative Endocrinology.

[ref-39] Le PP, Friedman JR, Schug J, Brestelli JE, Parker JB, Bochkis IM, Kaestner KH (2005). Glucocorticoid receptor-dependent gene regulatory networks. PLoS Genetics.

[ref-40] Levine S, Steckler T, Kalin N, Reul JMHM (2005). Stress: an historical perspective. Handbook of Stress and the Brain.

[ref-41] Lofts B, Marshall AJ (1960). The experimental regulation of the sexual cycle in the Brambling Fringilla montifringilla. Ibis.

[ref-42] Marino A, Baldi R (2008). Vigilance patterns of territorial guanacos (*Lama guanicoe*): the role of reproductive interests and predation risk. Ethology.

[ref-43] Marino A, Baldi R (2014). Ecological correlates of group-size variation in a resource-defense ungulate, the sedentary guanaco. PLoS ONE.

[ref-44] Marler P, Peters S, Ball GF, Dufty AM, Wingfield JC (1988). The role of sex steroids in the acquisition and production of birdsong. Nature.

[ref-45] Mateo JM, Cavigelli SA (2005). A validation of extraction methods for noninvasive sampling of glucocorticoids in free-living ground squirrels. Physiological and Biochemical Zoology.

[ref-46] Mattocks PW (1976). The role of gonadal hormones in the regulation of the premigratory fat deposition in the White-crowned Sparrow Zonotrichia leucophrys gambelii. Unpublished Master’s thesis.

[ref-47] McEwen BS, Wingfield JC (2003). What is in a name? Integrating homeostasis, allostasis and stress. Hormones and Behavior.

[ref-48] McGlothlin JW, Whittaker DJ, Schrock SE, Gerlach NM, Jawor JM, Snajdr EA, Ketterson ED (2010). Natural selection on testosterone production in a wild songbird population. The American Naturalist.

[ref-49] Moberg GP (1991). How behavioral stress disrupts the endocrine control of reproduction in domestic animals. Journal Dairy Science.

[ref-50] Montes MC, Carmanchahi PD, Rey AY, Funes MC (2005). Live shearing free-ranging guanacos (Lama guanicoe) in Patagonia for sustainable use. Journal of Arid Environments.

[ref-51] Mostl E, Palme R (2002). Hormones as indicators of stress. Domestic Animal Endocrinology.

[ref-52] Möstl E, Rettenbacher S, Palme R (2005). Measurement of corticosterone metabolites in birds’ droppings: an analytical approach. Annals of the New York Academy of Sciences.

[ref-53] Moyer KE (1968). Kinds of aggression and their physiological basis. Communications in Behavioral Biology.

[ref-54] Muller MN, Wrangham RW (2004). Dominance, aggression and testosterone in wild chimpanzees: a test of the dChallenge hypothesisT. Animal Behavior.

[ref-55] Naik DV, George JC (1963). Histochemical demonstration of increased corticoid level in the adrenal of *Sturnus roseus*(Linnaeus) towards the migratory phase. Pavo.

[ref-56] Nespolo RF, Bacigalupe LD, Bozinovic F (2003). Heritability of energetics in a wild mammal, the leaf eared –mouse (*Phyllotis darwini*). Evolution.

[ref-57] O’Reilly KM, Wingfield JC (1995). Spring and autumn migration in Arctic shorebirds: same distance, different strategies. American Zoologist.

[ref-58] Ostner J, Kappeler PM (2002). Seasonal variation and social correlates of androgen excretion in male redfronted lemurs (Eulemur fulvus rufus). Behavioral Ecology and Sociobiology.

[ref-59] Ovejero R (2013). Variación del nivel de cortisol en función de factores sociales y ambientales en guanacos (*Lama guanicoe*). Implicancias para la conservación y manejo de las poblaciones silvestres. Tesis doctoral..

[ref-60] Ovejero R, Acebes P, Malo JE, Traba J, Mosca Torres ME, Borghi CE (2011). Lack of feral livestock interference with native guanaco during the dry season in a South American desert. European Journal of Wildlife Research.

[ref-61] Ovejero R, Carmanchahi P (2012). Stress In Nature? Integrating Physiology, Ecology and Natural History of guanacos (*Lama guanicoe*).

[ref-62] Peczely P (1976). E’ tude circannuelle de la fonction corticosurrenalienne chez les espe ces de passereaux migrant et non migrant. General and Comparative Endocrinology.

[ref-63] Pereira RJG, Duarte JMB, Negrão JA (2006). Effects of environmental conditions, human activity, reproduction, antler cycle and grouping on fecal glucocorticoids of free-ranging Pampas deer stags (*Ozotoceros bezoarticus bezoarticus*). Hormones and Behavior.

[ref-64] Pride RE (2005). High faecal glucocorticoid levels predict mortality in ring-tailed lemurs (*Lemur catta*). Biological Letters.

[ref-65] Puig S, Rabinovich J, Puig S (1995). Abundancia y distribución de las poblaciones de guanacos. Capít ulo 4. Técnicas para el Manejo del Guanaco.

[ref-66] Puig S, Videla F, Puig) S (1995). Comportamiento y organización social del guanaco. Capít ulo 7. Técnicas para el Manejo del Guanaco.

[ref-67] Puig S, Videla F, Cona MI, Roig VG (2008). Habitat use by guanacos (Lama guanicoe, Camelidae) in northern Patagonia (Mendoza, Argentina). Studies on Neotropical Fauna and Environment.

[ref-68] R Development Core Team (2012). R: A language and environment for statistical computing.

[ref-69] Radovani N, Novaro A, Walker S, Funes M (2004). Parámetros poblacionales del guanaco (Lama guanicoe) en un área con actividad petrolera y cacería en Patagonia. Resúmenes de la II Reunión Binacional Argentino Chilena de Ecología.

[ref-70] Raedeke K (1979). Population dynamics and socioecology of the guanaco (Lama guanicoe) of Magallanes, Chile. Doctoral Dissertation.

[ref-71] Raouf SA, Smith LC, Brown MB, Wingfield JC, Brown CR (2006). Glucocorticoid hormone levels increase with group size and parasite load in cliff swallows. Animal Behavior.

[ref-72] Roff DA (1992). The evolution of life histories: theory and analysis.

[ref-73] Romero LM (2002). Seasonal changes in plasma glucocorticoid concentrations in free-living vertebrates. General and Comparative Endocrinology.

[ref-74] Romero LM (2004). Physiological stress in ecology: lessons from biomedical research. Trends in Ecology & Evolution.

[ref-75] Romero LM, Dickens MJ, Cyr NE (2009). The reactive scope model–a new model integrating homeostasis, allostasis, and stress. Hormones and Behavior.

[ref-76] Romero LM, Remage-Healey L (2000). Daily and seasonal variation in response to stress in captive starlings (Sturnus vulgaris): corticosterone. General and Comparative Endocrinology.

[ref-77] Rubenstein DR, Shen SF (2009). Reproductive conflict and the costs of social status in cooperatively breeding vertebrates. The American Naturalist.

[ref-78] Sapolsky RM, Becker JB, Breedlove SM, Crews D, McCarthy MM (2002). Endocrinology of the stress response. Behavioral endocrinology.

[ref-79] Sapolsky RM, Romero LM, Munck MU (2000). How do glucocorticoids influence stress responses? Integrating permissive, suppressive, stimulatory, and preparative actions. Endocrine Reviews.

[ref-80] Schradin C (2008). Seasonal changes in testosterone and corticosterone levels in four social classes of a desert dwelling sociable rodent. Hormones and Behavior.

[ref-81] Schwabl H, Wingfield JC, Farner DS (1984). Endocrine correlates of autumnal behavior in sedentary and migratory individuals of a partially migratory population of the european blackbird (Turdus merula). The Auk.

[ref-82] Sheriff MJ, Bosson CO, Krebs CJ, Boonstra R (2009). A noninvasive technique for measuring fecal cortisol metabolites in snowshoe hares (Lepus americanus). Journal of Comparative Physiology. B, Biochemical, Systemic, and Environmental Physiology.

[ref-83] Sheriff MJ, Dantzer B, Delehanty B, Palme R, Boonstra R (2011). Measuring stress in wildlife: techniques for quantifying glucocorticoids. Oecologia.

[ref-84] Sinervo B, Miles DB, Frankino WA, Klukowski M, DeNardo DF (2000). Testosterone, endurance, and Darwinian fitness: natural selection and sexual selection on the physiological bases of alternative male behaviors in side-Blotched lizards. Hormones and Behavior.

[ref-85] Smith JE, Monclús R, Wantuck D, Florant GL, Blumstein DT (2012). Fecal glucocorticoid metabolites in wild yellow-bellied marmots: experimental validation, individual differences and ecological correlates. General and Comparative Endocrinology.

[ref-86] Soto-Gamboa M, Gonzalez S, Hayes LD, Ebensperger L (2009). Validation of a radioimmunoassay for measuring fecal cortisol metabolites in the hystricomorph rodent *Octodon degus*. Journal of Experimental Zoology.

[ref-87] Soto-Gamboa M, Villalon M, Bozinovic F (2005). Social cues and hormone levels in male Octodon degus (Rodentia): a field test of the challenge hypothesis. Hormones and Behavior.

[ref-88] Stearns SC (1992). The evolution of life histories.

[ref-89] Taraborelli P, Ovejero R, Moreno P, Gregorio P, Schroeder NM, Torres Mosca ME, Carmanchahi P (2014). Different factors that modify anti-predator behaviour in guanacos (Lama guanicoe). Acta Theriologica.

[ref-90] Tarlow EM, Blumstein DT (2007). Evaluating methods to quantify anthropogenic stressors on wild animals. Applied Animal Behaviour Science.

[ref-91] Tempel DJ, Gutierrez RJ (2004). Factors related to fecal corticosterone levels in California Spotted Owls: implications for assessing chronic stress. Conservation Biology.

[ref-92] Touma C, Palme R (2005). Measuring fecal glucocorticoid metabolites in mammals and birds: the importance of validation. Annals of the New York Academy of Sciences.

[ref-93] Vera F, Zenuto RR, Antenucci CD (2012). Differential responses of cortisol and corticosterone to adrenocorticotropic hormone (ACTH) in a subterranean rodent (*Ctenomys talarum*). Journal of Experimental Zoology A.

[ref-94] Von Holst D (1998). The concept of stress and its relevance for animal behavior. Stress and Behavior.

[ref-95] Wasser SK, Hunt KE, Brown JL, Cooper K, Crockett CM, Bechert U, Millspaugh JJ, Larson S, Monfort SL (2000). A generalized fecal glucocorticoid assay for use in a diverse array of nondomestic mammalian and avian species. General and Comparative Endocrinology.

[ref-96] Wingfield JC (2005). The concept of allostasis: coping with a capricious environment. Journal of Mammalogy.

[ref-97] Wingfield JC, Hegner RE, Dufty Jr AM, Ball GF (1990). The “Challenge Hypothesis”: theoretical implications for patterns of testosterone secretion, mating systems, and breeding strategies. The American Naturalist.

[ref-98] Wingfield JC, Hunt K, Breuner C, Dunlap K, Fowler GS, Freed L, Lepson J, Clemmons JR, Buchholz R (1997). Environmental stress, field endocrinology, and conservation biology. Behavioral Approaches to Conservation in the Wild.

[ref-99] Wingfield JC, Sapolsky RM (2003). Reproduction and resistance to stress: when and how. Journal of Neuroendocrinology.

[ref-100] Wingfield JC, Silverin B (1986). Effects of corticosterone on territorial behavior of free-living male song sparrows Melospiza melodia. Hormones and Behavior.

[ref-101] Young AJ, Carlson AA, Monfort SL, Russell AF, Bennett NC, Clutton-Brock T (2006). Stress and the suppression of subordinate reproduction in cooperatively breeding meerkats. Proceedings of the National Academy of Sciences of the United States of America.

[ref-102] Young JK, Franklin WL (2004). Activity budget patterns in family-group and solitary territorial male guanacos. Revista Chilena De Historia Natural.

[ref-103] Zapata B, Gimpel J, Bonacic C, González B, Riveros JL, Ramirez A, Bas F, Macdonald D (2004). The effect of transport on cortisol, glucose, heart rate, leukocutes and weight in cautive-reared guanacos (Lama guanicoe). Animal Welfare.

[ref-104] Zera AJ, Harshman LG (2001). The physiology of life history trade-offs in animals. Annual Review of Ecology and Systematics.

